# The exploration of consciousness in insects

**DOI:** 10.1098/rstb.2024.0302

**Published:** 2025-11-13

**Authors:** Lars Chittka, Sarah Skeels, Olga Dyakova, Maxime Janbon

**Affiliations:** ^1^ School of Biological and Behavioural Sciences, Queen Mary University of London, London E1 4NS, UK

**Keywords:** bees, cognition, emotion, flies, prediction, selfhood, sleep

## Abstract

Consciousness is a state of subjective experience or awareness, e.g. of an emotion, the self or external objects. In humans, this awareness is underpinned by a suite of cognitive functions, from attention to metacognition. To understand the evolution of consciousness, the study of these cognitive functions across a variety of animal taxa is critical. Insects are useful organisms because we have a sophisticated understanding of their cognition from over a century of study and modern tools are revealing the intricacies of insect brains with increasing clarity. Here we cover the rich history of this venerable field as well as more recent discoveries relating to consciousness in insects, specifically focusing on the following areas: emotions, the distinction of self and other, prediction, attention and active sleep. There can still be no formal certainty about consciousness in insects; even in humans, there is currently no agreement over the particular combination of cognition and neural function that produces consciousness. Nonetheless, evidence from all the lines of investigation summarized here builds up to an increasing probability that insects might possess some form of subjective experience. We encourage further investigation of insects to explore the building blocks of consciousness and its evolutionary antecedents.

This article is part of the theme issue ‘Evolutionary functions of consciousness’.

## 1. Introduction

Consciousness comprises many interrelated phenomena, e.g. the capacities to feel, experience and think. It may have evolved as a consequence of building a unified simulation of the organism within its environment [1–4]. Such a simulation might afford an organism important advantages, such as a greater ability to navigate an environment, plan for the future or respond to unexpected conditions. Around the beginning of the 20th century, insects were seen as an early test case for the exploration of consciousness in animals.

A remarkable example is the 1902 essay (in German) entitled ‘The psychological abilities of ants and some other insects’ by the Swiss psychiatrist Auguste Forel [5] (one of the founding fathers of neuron theory). Forel rejected the dualism of body and mind and pointed out that consciousness and other psychological processes cannot be understood without exploring the underpinning neural processes: ‘Among the phenomena of our brain-life, as wonderful as they are, lies absolutely nothing contrary to the laws of nature, or that would require invocation of a mystical, supernatural ’psyche’ [5]. Forel held that true consciousness is the ability to introspect (equivalent to the contemporary definition of higher-order consciousness [6]) and proposed that this capacity is reserved for humans. He thought that a more basic form of mind comprises the processes that in humans are *accessible by introspection*, which he described as ‘the objects of attention: perceptual images, impulses of will, feelings or abstract thought’ [5]. Forel highlighted that direct access to conscious experience is only available in oneself, and that to the extent that we can use arguments of analogy about consciousness in fellow human beings, we can logically also extrapolate to other animals [5]. Our review is inspired by this approach, and we explore several of Forel’s ‘objects of attention’ in this article.

Following Buttel-Reepen [7] and Wasmann [8], Forel firmly rejected the (then popular) claim that insects are reflex machines [7,8]. He explained (with reference to the human brain) that while sensory input, motor control, attention and emotion are all underpinned by separate neural pathways, they are tightly interfaced in the brain; he further highlighted that motor output can result in emotions such as pleasure and pain, reinforcing certain behaviours but not others [5]. He provided evidence that Hymenopteran insects (e.g. bees and ants) also integrate multiple sensory memories into percepts and suggested that such interfacing is probably phylogenetically ancient and thus common to many animals. He listed experimental evidence for memory in many insects, and suspected that it is present in all of them [5]. Contrary to Bethe [9] (who denied the existence of memory in insects, but curiously thought that such memory would be evidence for consciousness, if it did exist), Forel [5] (as well as Buttel-Reepen [7] and Wasmann [8]) cautioned that associative memory is insufficient evidence for consciousness [9]. This century-old debate recently resurfaced when it was proposed that consciousness arises from and is functionally tied to associative learning [10]. We agree with Forel and his contemporaries that, at least in its basic form, associative learning falls significantly short of the criteria required for the diagnosis of consciousness.

Using experiments with artificial flowers in bees, Forel provided evidence for top–down attention, showing that bees trained to one type of flower are difficult to distract by other flower designs, even familiar ones [5]. He discussed the presence of a will in ants, which he observed battling with obstacles for hours, when they knew the value of an out-of-reach item (see also Turner [11] for the discussion of a will in cockroaches). Forel described the emotion-like components of winner–loser effects in ants. After a battle with a competing ant colony, the losing party falls into a state of ‘hopeless abandon’ (more so if they lost multiple battles), making them less likely to engage in or win further battles and resulting in ‘cowardly retreat’, consumption of own brood, and neglect of work [5]. Conversely, winning a battle, in Forel’s observations, promotes courage and tenacity in the superior party, which might make them succeed in attacking even physically superior enemies [5]. To our knowledge, this is not just the first description of winner–loser effects in any animal, but also a visionary exploration of emotion-like states in this context. Forel highlights that such states can affect multiple other behaviours in the aftermath of a salient event.

Citing examples from bees and ants, Forel highlighted the importance of conflicts between various perceptions, emotions and efforts of the will in the study of animal minds: for example, the question of how animals balance conflicting needs such as the motivation to feed and colony defence. He placed particular emphasis on cases where stimuli are not concurrently accessible and must therefore be based on mental representations. Interestingly, such motivational tradeoffs have more recently been explored as indicators of animal sentience (the capacity to feel) and specifically pain experiences [12,13]. More broadly, Forel pointed to the likely deep evolutionary origins of emotions and highlighted that instinct and emotions are deeply intertwined, for example in brood care.

Forel and his contemporaries were by no means uncritical—they repeatedly warned against the risks of anthropocentrism, and against setting the bar for criteria of consciousness too low [5,7,8]. They realized that the indicators they provided were fragmentary and tentative, and that while formal proof of mental processes in animals was impossible to obtain, a useful alternative was to search for likely *signs* of such processes, and to combine indicators from multiple lines of investigation for an assessment of the probability that an animal is conscious. Buttel-Reepen ultimately concluded that bees have either no consciousness, or one of the lowest degree [14]. His criteria were even more exclusive than Forel’s [5]; Buttel-Reepen posited that unity and continuity of conscious experience, the integration of all sensory experiences into a cohesive whole, and power of imagination were necessary ingredients that needed to be demonstrated. Buttel-Reepen doubted that these were present in insects, but was open to the possibility of sentience: he encouraged the exploration of bee-specific emotion-like states such as might occur during swarming, where he found bees to display ‘a bacchantic delight’ and wrote that ‘it is as if the bees were indeed drunk with joy’ [7]. Like Forel [5], Turner [11] and Wasmann [8], he provided clear evidence for memory and touched upon many of the theoretical approaches and experimental paradigms that prevail today in the study of the relation between consciousness, attention, emotion and cognition [7,14]. In 1908, summarizing this literature and taking a broader perspective across animals (including both vertebrates and invertebrates), the pioneering animal consciousness researcher Margaret Washburn pointed out the tight links between action and perception, and between motor behaviour and consciousness [15]. In this manner she not only prefigured key ideas of the predictive processing theory of consciousness [16,17], but she also pointed out that the study of sleep (including both non-conscious phases and conscious dream phases) might be a useful avenue of research into the very nature of consciousness [18]. We return to this approach later in this review.

Unfortunately, the progressive ideas of this school were all but buried under the fashion of behaviourism. Many animal psychologists favoured the easier approach to use directly observable behaviour to understand cognition [19], often ridiculing the efforts of those interested in mental processes [20]. As an isolated example to the contrary, Charles Turner, an African-American pioneer of animal cognition, continued to point out the likely rich inner world of insects until his death in 1929 [21,22]. He directly confronted Edward Thorndike (one of the founding fathers of behaviourism), highlighting that the navigation behaviour of homing wasps could not be explained by trial-and-error learning but must involve a form of intentionality [23]. Regrettably, Turner’s visionary contributions were largely forgotten, as were those from the other early explorers of insect minds.

A curious result is that insect scientists studying mental processes today, even though their forebears had pioneered the field around the penultimate turn of the century, find themselves on the back foot when comparing their work on the topic with scientists exploring similar questions in vertebrates. When Donald Griffin returned to the question of animal consciousness in the 1970s, he faced extensive criticism [24,25]. Even though Griffin included the insects in his discussions, the field now restarted with a focus on large-brained vertebrates [26–29].

We here follow Forel’s [5] early approach of searching for indicators of the probability of consciousness in animals rather than formal certainty. This is mirrored by recent trends of breaking consciousness down into its constituent parts or along several dimensions [30,31]. We draw on both schools of thought to ask whether analogues of vertebrate consciousness might be present in insects. Our review is not comprehensive—we focus specifically on domains of consciousness in insects that have received less coverage in recent review articles than others. These are emotion-like states and selfhood, prediction and attention, and the relationship between sleep and consciousness.

## 2. Emotion-like states

One of the key ingredients of consciousness is the capacity to have affective states, which include emotions and moods. The question of whether insects have an emotional life is no less (and no more) difficult to answer than in other non-human animals. This is because insects, like other animals, cannot verbally report their emotions, if they have any. The perception that emotions might be more easily studied in animals with closer phylogenetic distance to humans, or more similar brain structures, might sometimes be misleading. It is simply impossible to infer function from gross neuroanatomy, even in closely related species: for example, chimpanzees have Broca’s and Wernicke’s areas (which in humans are used for speech production and comprehension), but chimps demonstrably lack spoken language [32]. Likewise, the view that facial expressions of humans’ mammalian relatives offer a more direct window into their emotional world is tempting, but often subject to anthropomorphic misinterpretation [33,34]. Nonetheless, insect scientists often find themselves confronted with disproportionally high levels of scepticism when exploring emotion-like states [5,35].

**Figure 1.**
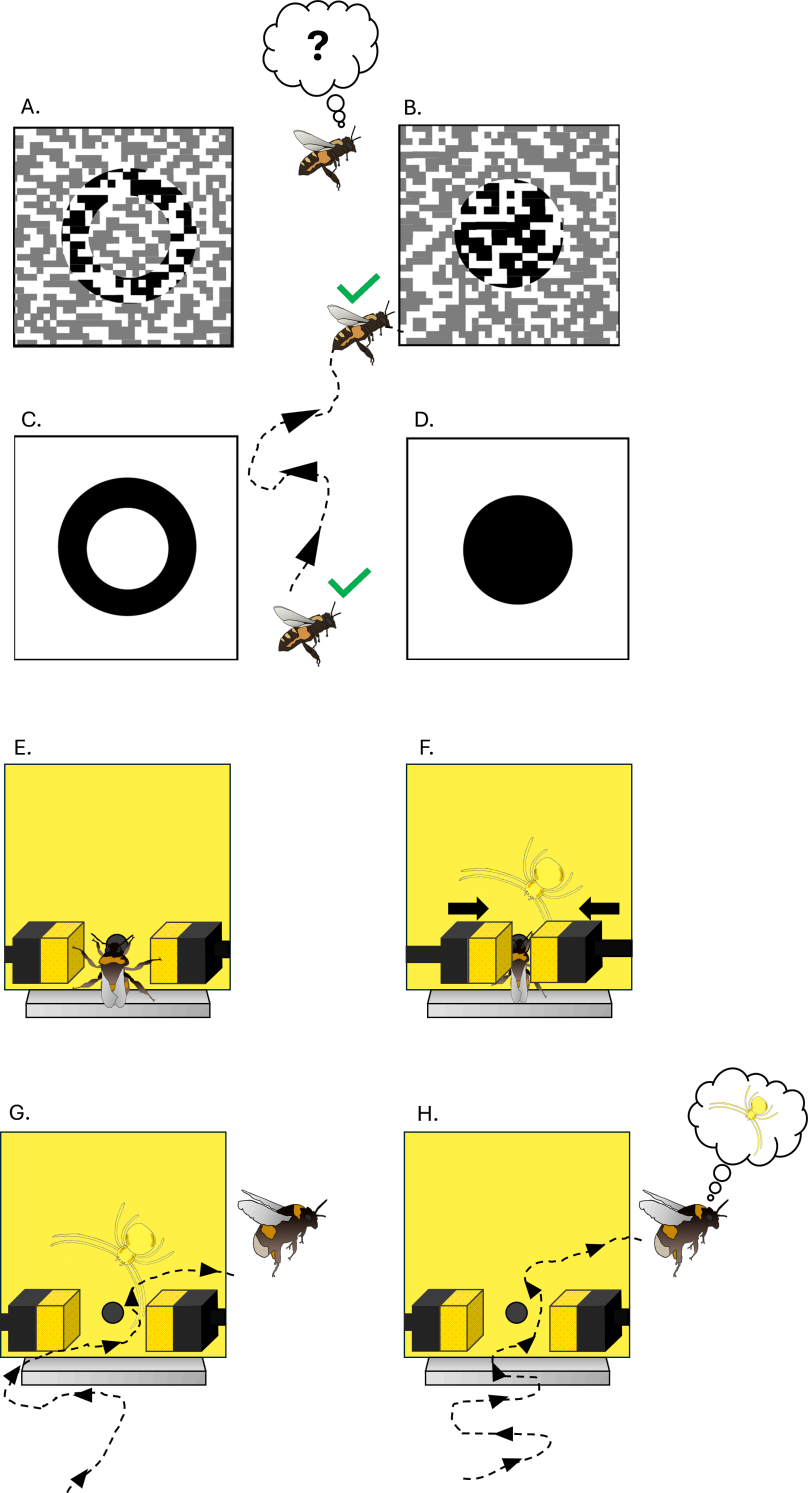
Attention and false alarms in bees. (A–D) Honeybees fail to learn to distinguish the two camouflaged shapes in panels (A) and (B) even after extensive training. However, the task can be introduced by using non-camouflaged targets (C) and (D), guiding bees to know what to look out for. If bees so entrained are then tested with the camouflaged objects in (A) and (B), they discriminate them with high accuracy (after [28] modified). (E–H) Bumblebees trained to avoid robotic crab spiders subsequently display false alarms as if they had ‘hallucinated’ a spider [29]. Bumblebees are trained to forage in an array of artificial ‘flowers‘ (yellow squares; E–H), a minority of which harbour plastic models of predatory, cryptic crab spiders (F). If bees land on such flowers during training, they are briefly trapped by solenoid-driven, sponge-clad pincers to simulate a predation attempt (F). During later testing, bees display hesitation and scanning behaviour at all flowers, and will typically reject flowers with spider models (correct–reject, (G)). However, while bees land on the majority of safe flowers, they also display elevated levels of false alarms (H), where they behave as if they imagined the presence of a spider.

Consider the following examples from various insect taxa. (i) Male fruit flies that are stressed by being deprived of mating opportunities seek out alcohol [36]. (ii) The perception of dead conspecifics by fruit flies alters sensory perception and brain physiology, and the effects are so profound that they reduce longevity [37]. (iii) Again in fruit flies, it was found that painful stimuli can induce a heightened state of vigilance and a hypersensitivity to previously non-painful stimuli, similarly to what is observed in humans with chronic pain conditions [38]. (iv) Bumblebees, after learning to avoid cryptic crab spiders on flowers by being exposed to simulated attacks, display false alarms—rejecting perfectly safe flowers after inspecting them, as if they had experienced a ‘hallucination’ of a spider (figure 1E–H) [39]. (v) In crickets, repeated defeats in male–male contests of crickets induce a long-term depression-like behaviour state, but crickets that received drugs affecting the serotonin system showed higher resilience to chronic defeat stress [40]; see also [41–43] for related work on other insects, including the remedying effects of antidepressants.

Transposing these scenarios to the human realm, it seems obvious that they have significant emotional components, and that this is so because of their fitness relevance. The emotional element in such situations provides a motivation to avoid unfavourable conditions; the seeking of drugs (such as alcohol) occurs because basically, they make one *feel* good, especially when one is miserable [44,45].

When rodents or other mammals are trained to associate a (previously neutral) stimulus with an aversive one, e.g. an electric shock, then this is commonly referred to as fear conditioning [46]. The conditioned stimulus subsequently triggers avoidance, immobility (‘freezing’), startle and escape responses, changes in heart rate and breathing, as well as recognizable activity changes in brain areas such as the amygdala [46,47]. It is implied that the affective fear response is a crucial, survival-related ingredient in the learning process [48].

Insects such as fruit flies (including their larvae) [49–53] and bees [54–56] also learn to associate conditioned stimuli (e.g. odours, visual stimuli) with aversive stimuli; crucially, similar types of overt behaviour as listed for mammals (above) have also been found in insects [39,57], as well as changes in breathing [58] and heart rate [57] and the expression of stress-related genes [56]. The evidence for an affective component in the responses to noxious stimuli has been reviewed in detail elsewhere [59,60]. It has been suggested that the link between various mushroom body neuron types in fruit flies performs a similar function as the amygdala–hippocampus axis in mammals in the context of the learning of aversive stimuli [61]. Despite these many similarities between mammals and insects, in the latter such learning is commonly referred to as ‘just’ classical conditioning, without an affective component.

**Figure 2.**
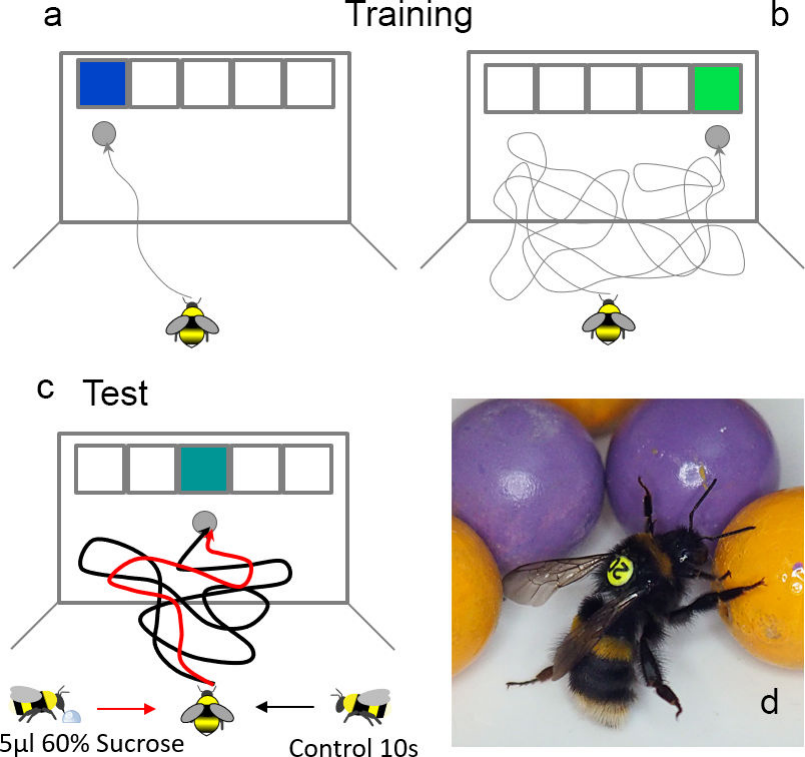
Emotion-like states in bees. (a–c) A positive cognitive bias in bees [63]. Bumble bees familiarize themselves with a flight arena in which, on the back wall, the blue target on the left always contains a droplet of sugar water (a), whereas the green target on the right does not (b). Only one of the five positions on the choice wall is ever accessible. After training, bees will usually approach the blue target (left) in a straight line when it is available (a). Conversely, hesitation is apparent if the green target (right) is on display (b). When intermediate (‘ambiguous’) turquoise stimuli (centre) are offered (c), bees will hesitate for a length of time that lies between the flight times displayed for blue and green (black flight trajectory). If, however, a tiny drop of sucrose solution (5 μl) is offered as a surprise before bees enter the experiment, they judge the ambiguous option (turquoise) as more promising and will accept it more swiftly (red flight trajectory)—i.e. they judge the intermediate stimulus in a more optimistic manner than control bees (figure by Cwyn Solvi, printed here with permission). (d) Do bumble bees play [64]? When given the opportunity to roll balls in the vicinity of the nest, bumblebees will roll balls for extended periods, even if no appetitive reward is made available near the balls. As opposed to rewardless flowers (which are only probed a few times before bees give up), bees return repeatedly to this activity, even increasing ball-rolling behaviour over time, indicating an enjoyment of the activity itself, rather than a form of food search exploration. Young bees engaged with the balls more than older bees. Photo by Richard Rickitt (with permission).

Some tests of emotions in insects have been modelled directly on vertebrate paradigms and should therefore be comparable. So-called cognitive bias tests have been developed to test the psychological welfare of animals (e.g. rats) in captivity [62]. These paradigms are essentially versions of the proverbial glass that can be half-full or half-empty: optimistic humans might view the same ambiguous state (a glass filled to the middle with a drink) as positive, whereas a pessimist would judge the glass as being nearly empty. Bees respond to ambiguous stimuli with more reservation when they have previously suffered a simulated predator attack, and more positively if they had experienced a surprise reward before the experiment—a dopamine-dependent, optimism-like state (figure 2a–c) [63,65]. It has been suggested that such results could alternatively be explained by motivation, although this simply replaces one emotion-related explanation with another: motivation is itself a mental state, not an observable or quantifiable behaviour or a simple mechanism [66]. In recent work, the question of whether insect responses to ambiguous stimuli could be explained by cognitive biases, motivation or altered attentional states was directly addressed, and it was still found that emotion-like cognitive biases are the most likely explanation [67].

Play-like behaviour was also found in insects. When offered small coloured balls in an arena near their nest, bees rolled the balls for extended periods even when no sugar reward was made available (figure 2d) [64]. Bees went out of their way to return repeatedly to such a ‘play area’. There seemed to be something inherently rewarding in the activity itself, since no other incentive was offered. In line with what is commonly observed in vertebrate play behaviour, young bees engaged more often with the balls than older ones. Play-like behaviour was also recently discovered in fruit flies, where some individuals appear to enjoy the ‘thrill’ of riding a carousel. Such self-stimulation was proposed to aid self-perception through the training of internal models [68].

We cannot definitely know what (if anything) an insect feels under such conditions (any or more or less than what we can infer about a mouse). Indeed, some seemingly emotion-related insect behaviours might in some cases have explanations that do not involve sentience. A useful test case is the condition of learned helplessness—a psychological state that occurs after repeated exposure to inescapable aversive stimuli. Many animals— including insects—subsequently endure such stimuli without trying to flee, even if the possibility to escape is again made available [69]. Even though this state looks overtly similar to what humans might experience as hopelessness, the state can be induced in rats with severed spines [69] and in decapitated cockroaches [70]. This shows that what looks like a ‘miserable’ condition is not necessarily evidence for sentience [69]. However, it is unlikely that antidepressants alleviate the state of learned helplessness in animals with a disconnected brain, as they do in some insects with intact bodies [40,42].

A common response to observations of what appear to be emotion-like states in insects is that they might be manifestations of ‘just instinct’ [71]. That could certainly be the case, though it is not an alternative explanation, but an additional one. An alien observer of human behaviour might conclude that parental love, rage, sexual desire, grief, etc. are just instinct—and they would be correct. While all of these might be subject to cultural variation and individual experience, they are part of our default emotional toolkit [5]—the human affectome [72]. Examples where emotions serve ultimate functions are the enhancement of learning, the promotion of parent–offspring care and attachment, and the avoidance of dangerous situations. All of these are fitness-related and at least in part instinctual in humans, and with some probability, non-human animals [73–75]. Thus, even if emotions are innate, they are nonetheless important ingredients of an animal’s subjective experience.

## 3. Self and other

One important aspect of consciousness is self-recognition, which at its core requires an individual to be able to recognize itself as distinct from another living entity [31,76,77]. At the start of the 20th century, Margaret Washburn [15] proposed that motor behaviour is at the heart of consciousness: rather than just responding to incoming stimuli, animals constantly probe their environment and must distinguish between self-generated and other-generated sensory input [15]. The transition from a sedentary lifestyle to locomotion early in evolution would already have required animals to make this distinction [77]. Animals create an internal copy of the self-generated signal, known as an ‘efference copy’, to cancel out the feedback from their own movements [78,79]. Consider the scenario where a fist suddenly appears in someone’s visual field. This is no cause for alarm if they have just willingly raised their hand to inspect their knuckles—but if they have not, and it is someone else’s fist, then they could be under attack. The visual stimulus might be similar, but it is the presence or absence of the efference copy of the intended movement that allows the person to distinguish between the two, and then respond accordingly.

Body awareness is considered an important aspect of self-awareness and individual experience [80]. For example, an animal’s extremities constitute perfectly palatable food, yet animals are not normally inclined to consume their own legs, since there is some form of appreciation of own body integrity—what is part of *me* and what is not [81]. Some ant species (such as *Cataglyphis bicolor*) show remarkable rescue behaviour of nestmates. When a nestmate is experimentally trapped under rubble (using a nylon tether around the ant’s waist), rescuers will come to their aid. These helpers show appreciation of the trapped ant’s body dimensions by removing rubble only from above buried (non-visible) body parts. They bite forcefully at the tether, but only gently pull on legs of the trapped ants [82]. These behaviours often involve multiple rescuers, meaning that there will be occurrences where individuals will need to discern which extremity is part of themselves and which parts belong to another rescuer and/or the trapped ant [83]. As such, body awareness would be important here. Whether empathy is involved in such rescuing, as has been suggested for similar behaviour in rodents, remains an open question [84].

**Figure 3.**
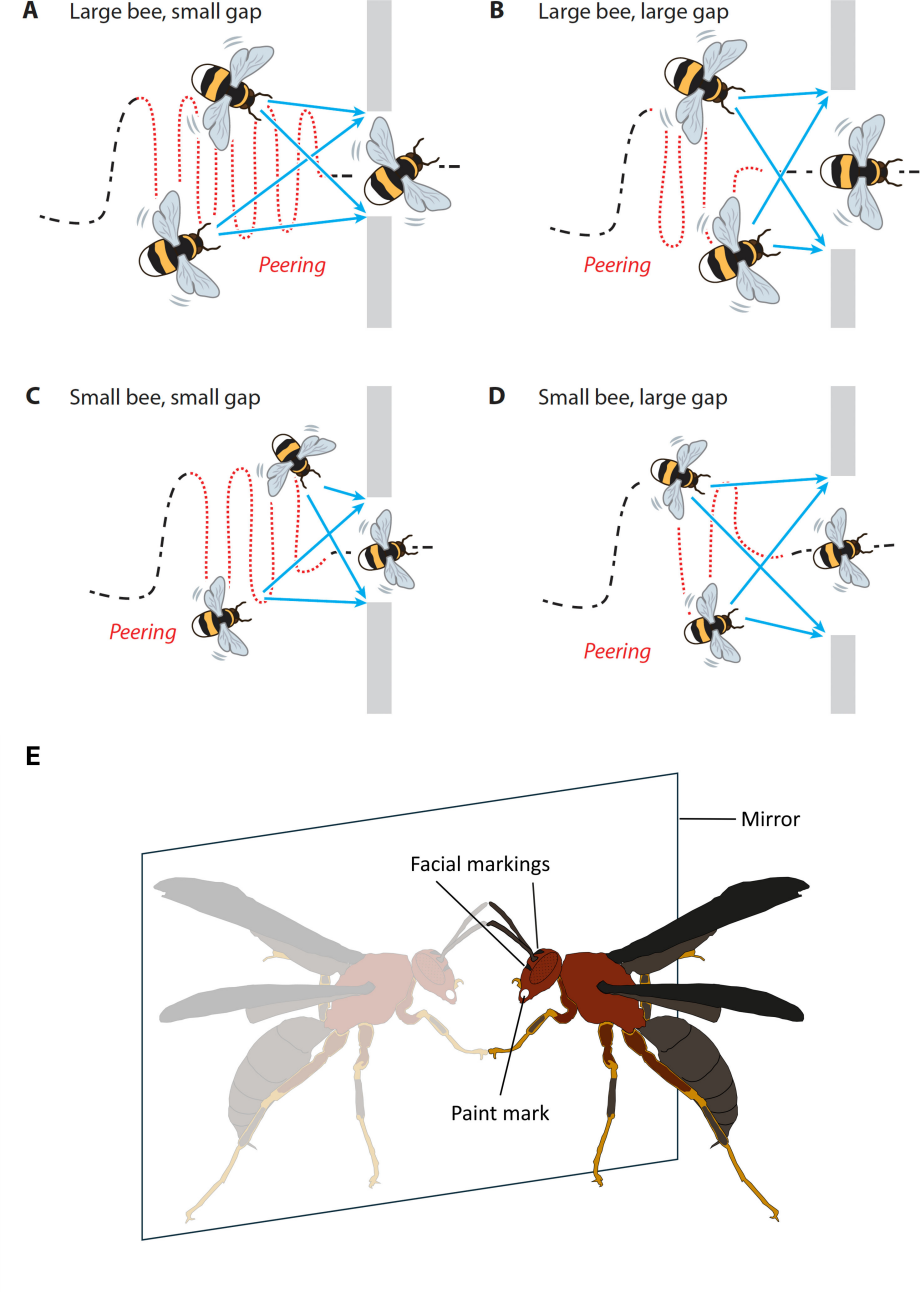
Self-recognition in insects? (A–D) Bumblebees of the same species are highly variable in body dimensions and appear to know their own body size, as evidenced from behaviour when navigating differently sized gaps. Bees will approach and inspect the gap from different angles to work out its size. Larger bees will peer more when traversing a narrow gap compared with their smaller conspecifics (A, C). Bees will angle their body position to avoid collision if the gap is similar to or narrower than their wingspan (A). Figure from [80], after [85]. (E) Individuals of *Polistes metricus* paper wasps have distinct face marks, allowing for individual recognition of nest mates. Mirror tests were used to investigate the possibility of self-recognition of individuals with paint marks on their faces. Visible marks elicited more exploratory behaviours (antennation and touching of the mirror) compared with controls with transparent paint marks. However, there was less self-directed grooming of the mark than expected, potentially indicating that wasps interpreted their mirror image as a previously unknown individual rather than a reflection of themselves [86].

Being able to take into account one’s own body size and movements when making decisions has obvious functional advantages when navigating a cluttered environment. Ravi *et al.* [85] found that flying bumblebees, which vary significantly in body size, were able to judge the size of a gap in a wall in relation to their own body size. Larger bees avoided the small gaps that were accepted by their smaller conspecifics, and when the size of the gap was borderline navigable for a bee of a given size, the bee would adjust her flight behaviour to minimize the risk of collision (and therefore injury) (figure 3A–D). Similarly, Ben-Nun *et al.* [87] demonstrated that self body-size perception in locusts is correlated with their own body size.

A popular test of self-recognition in vertebrates is the so-called ‘mirror and mark test’ [88,89]. The test involves putting a mark on the animal in a place that can only be seen in the mirror. Attempts to engage with and remove the mark from the body when in front of the mirror are interpreted to mean that an individual appreciates that their own individual appearance has been altered, thus showing a form of self-awareness [86,90,91]. The mirror test has been adapted to examine self-recognition in *Myrmica* ants [92]. Ants reportedly passed the test, but results are hard to interpret owing to the lack of appropriate controls. More promising study systems are certain species of *Polistes* wasps, since individual face recognition is part of these species’ social architecture [93].

Mirror self-recognition can be viewed as a special case of individual recognition. In species where individuals are not visually distinguishable by the visual–spatial resolution that their eyes permit, mirror self-recognition tests are less likely to produce positive results (though see below for a possibility via active vision). While *Polistes* wasps do respond to either the presence of mirrors or presence of marks placed on their faces by experimenters when seen in mirrors, there was no evidence that grooming was directed towards the mark [86]. Instead, wasps touched the mirror more when a visible mark had been placed on their faces than in a control condition using a clear (non-visible) mark (figure 3E), making it hard to know whether the behaviour was in any way self-directed, or whether the perception of what was seen in the mirror was that of an unfamiliar conspecific. It is thus possible that what they perceived in the mirror was not themselves, but an unfamiliar conspecific.

This highlights the ongoing debate about the validity of the mirror test and its capacity to show true self-awareness—or the absence of such awareness when results are negative [94]. If indeed mirror-self recognition is an extension of individual visual recognition in a social system, then it is important to consider that natural environments do not contain mirrors. No animal can be expected to comprehend spontaneously the workings of a mirror, so animals must be given ample opportunity to interact with mirrors to familiarize themselves with their own appearance before tests can commence. In doing so, they most probably figure out that their own actions result in fully predictable ‘actions’ of their mirror image [95,96]—which is a special case of animacy perception. In that sense, it is not clear why a mark is required: the animal that loses interest in the mirror after such an exploratory phase—and that ceases to treat their mirror image as another conspecific—might already have shown a form self-recognition. It is important to interrogate self-recognition (and self-awareness more broadly) in paradigms more relevant to the natural ecology of the animal being studied [97]. In some animals, different sensory modalities might be worth exploring for self-recognition tests. Some ants, for example, use olfactory cues for individual recognition [98]. In such species, chemosensory self-recognition tests might be more appropriate [99,100].

Metacognition, defined as ‘knowledge about one’s own knowledge’ [101], is another ingredient of self-recognition. It is often considered as an indicator of higher-order consciousness—the awareness of own mental states, rather than just experiencing the mental states themselves [102]. This is the form of true consciousness that Forel thought in 1902 might be reserved for humans [5], where indeed it has been extensively studied [103], but other primates have also been tested (see review by [104]). Perry & Barron [105] attempted to examine whether Western honeybees (*Apis mellifera*) have basic metacognition. Bees were given visual discrimination tasks that varied in difficulty and were rewarded with sucrose for correct choices but punished with bitter quinine for mistakes. Additionally, they had the option to leave the task. Perry & Barron [105] found that individuals left the task more often when the task was difficult, and that by doing this, they improved their success rate. They were also able to pass on this ‘opt out’ rule to a novel task. These results suggest that the bees were able to assess their certainty on a given trial and make decisions accordingly, indicating the possibility of some kind of metacognitive processing [105,106]. However, it is also possible that their choices were based on a series of learnt associations [105–107] (see also [102] for equally mixed evidence in ants). Further work on metacognition and decision confidence is required for insects, perhaps more closely modelled on tasks commonly deployed in mammals [108–110]. Whether the cross-individual version of metacognition—namely, theory of mind (knowledge about information held by other animals)—exists in insects is presently unknown. Such ‘mental perspective taking’ is perhaps most rewardingly explored in settings where such an ability would convey clear fitness benefits. Examples are prey taking into account what predators might see (or *vice versa*) or animals strategically positioning themselves to amplify mating signals, depending on where they know audiences to be.

## 4. Prediction and attention

Many textbooks still perpetuate the narrative that brains are designed to respond to incoming stimuli, process information and then generate adaptive behaviour. However, during wakefulness, brains (and by extension, the behaving animals that house them) constantly probe their environment, moving sensors (e.g. eyes, extremities with touch sensors, etc.) to establish contingencies between behaviour and its outcome. Controlled variability in spontaneous nervous system activity promotes behavioural flexibility and active information acquisition, and it may result in flexible problem-solving [111,112]. In addition to the active moving of sensors, attention provides a sort of ‘inner eye’ to focus selectively on salient information from the sensory periphery. The role of attention has both been linked to consciousness and described as the process assigning greater precision to certain sensory streams [5,15,113].

As scholars surmised around the turn of the 20th century, there is probably a link between top–down, selective attention and consciousness [5,15]. To make sense of the environment, the awake brain constantly makes predictions about what sensory information to expect next [114]. These expectations are encoded across the brain as inner generative models, which are iteratively adjusted to minimize the discrepancy between predictions and sensory returns—or ‘prediction errors’ [115,116]. ‘Prediction error’ is actually a misnomer; it does not involve a mistake by the brain, just a violation of an expectation—a surprise. Prediction errors allow animals to adapt perception and behaviour based on a mismatch between expectation and input. An obvious type of predictive coding is foreseeing what changes in sensory input are expected as a result of the voluntary movements of sensors (see §3). In humans, when sensory inputs match the brain’s predictions, perception occurs without much conscious awareness. However, when there is a prediction error, heightened top–down attention can bring novel or unexpected stimuli into conscious awareness. This process can be essential for survival, since it probably facilitates adequate responses to specific threats or opportunities in the environment [31,117,118].

Insect perception and attention can also be understood under a predictive processing framework [119–122]. The innate preferences of a pollinator, for example, contain certain ‘default predictions’: e.g. naive bees expect flowers coloured violet to blue to be highly rewarding since flowers of these colours indeed contain better-quality rewards in some habitats [123]. Such ‘priors’ can be adjusted later by experience: an individual bee might learn that a plant species with yellow flowers is rewarding, and update its expectations accordingly. Bees searching for flowers while avoiding familiar predators do not just passively respond to objects that appear in their visual field. Instead, they ‘know what to look for’, and search specifically for memorized flower targets [124], as well as scanning for predators that they have seen before. Like humans, honeybees initially fail in detecting camouflaged shapes, but when the shapes are first presented openly, attention subsequently helps bees to find them in the camouflaged presentation (figure 1A–D) [125]. Bumblebees experienced with attacks from cryptic flower predators subsequently display false alarms—they reject many perfectly safe flowers as if they had ‘hallucinated’ the visual appearance of a predator (figure 1E–H) [39].

In humans, various levels of consciousness and attention are accompanied by characteristic oscillations of neural activity in the brain [126,127]. The awake human brain shows a characteristic 30 Hz frequency in EEG recordings; such *beta* oscillations, synchronized across cortical areas, are crucial in sensory awareness and focusing attention [128]. Insects display similar patterns: synchronization of oscillatory activity (at 18 Hz) across brain areas was found in awake honeybees [129]. Oscillations of 20−30 Hz also occur in the central complex of the brains of fruit flies when they attend to visual features of novel objects. Theta oscillations (approx. 4−8 Hz), which are implicated in memory, navigation and the coordination of top–down and bottom–up signals in humans [130,131], are also present in insect brains, particularly in the central complex and mushroom bodies [132]. These oscillations may serve similar roles in regulating temporal aspects of attention and memory. Furthermore, similar to humans [133,134], prediction errors are also distributed along the entire fly sensory processing hierarchy, enabling the rich interplay of predictions and prediction errors that could underpin conscious awareness [135,136]. So far, only *local* prediction errors have been tested in fruit flies—for example, a series of light flashes of one colour, followed by a so-called *oddball* with a different colour [135]. In human hearing, such oddballs elicit appreciable prediction error signals in the nervous system (limited to sensory cortical areas)—but this occurs even in unconscious human subjects [137]. Global oddballs, conversely, violate a previously learnt *pattern—*e.g. if tone strings CCCA CCCA CCCA are followed by CCCC. The arising *mismatch negativity* signal in higher-order brain areas of humans in this case is linked to conscious awareness [137,138]. Future work in the context of prediction errors in insects should focus specifically on the distinction between local and global deviations from expected patterns, and their neural underpinnings.

Prediction error-mediated surprises can result in emotion-like responses and in redirecting attention—e.g. bees finding rewards in an unexpected location exhibit a dopamine-dependent optimistic state [63]. In bees placed in a virtual reality environment, distinct neural activity in the optic lobes reliably precedes the selection of one or another stimulus to which subjects pay attention [139]. The existence of similar types of brainwaves in humans and insects is not formal proof of consciousness in the latter. However, it is at least suggestive that the type of synchronization across brain areas that, in humans, accompanies states of wakefulness and attention, also occurs in insects [71,132,140].

## 5. Active sleep, deep sleep and wakefulness

Everyone knows what is meant when it is said that a man is unconscious. He is neither awake, aware of the sights and sounds of the outer world, nor dreaming, aware of images that are the product of his own fancy. Consciousness is that which is present when we are either awake or dreaming, and which is absent when we are dreamlessly asleep.

Margaret Floy Washburn, 1916 [18]

Given the obvious division between conscious and non-conscious states that we experience daily, one useful entry point into the question of consciousness and its function in animals is to contrast it with what happens during sleep—which, perhaps counterintuitively, may be tightly related to the maintenance of consciousness in the wakeful state. One might expect that if there are no signals from the sensory periphery (such as when one’s eyes are closed during sleep), the brain might automatically be in a resting state as well. However, brains—whether insects’ or humans’—are never ‘switched off’, not even during the deepest sleep. In humans, sleep is divided into multiple phases: slow wave (‘deep’) sleep, and rapid eye movement (REM) sleep, which alternate in multiple phases every night [141]. Most dreams occur during REM sleep, and many of them are consciously experienced while the brain is largely ‘disconnected’ from the outside world [142]. Cortical activity during REM sleep resembles waking activity [143,144]. REM sleep is associated with emotional regulation and memory consolidation [142,145,146]. How this is achieved during REM sleep has been discussed under the predictive processing framework: a role for REM sleep might be to prune and refine internal generative models [147–149]. Such models might over time recruit a large portion of the brain’s resources as well as be overtrained on the particular external stimuli encountered, limiting the generalizability of predictions. In this context, a role for REM sleep might be that it acts as a closed-loop simulation, where, away from the demands of immediately reacting to the environment, imagined stimuli are processed to prune and refine the generative models and avoid neural overfitting. In this way, not only would the brain make more flexible predictions when awake, but the synaptic cost of maintaining such generative models might be reduced.

In insects such as honeybees, fruit flies and mosquitoes, sleep can be behaviourally diagnosed by quiescence (or immobility), increased arousal thresholds and sleep rebound (increased period of quiescence after sleep deprivation) [150–155]. Insects also have distinct sleep phases, although these may not map directly onto human ones: bees, for example, have three distinct sleep phases. Their deepest sleep is characterized by a distinctive crouching posture in which head, thorax, and abdomen are relaxed, antennae are immobile, muscle tonus and body temperature are decreased, and response thresholds to external stimuli are increased [151,156]. Exposing bees to odours during this sleep phase functions to consolidate experiences from the previous day in honey bees’ memory, whereas in humans, it is REM sleep that appears to be related to memory consolidation [157,158].

Fruit flies, too, have distinct stages of quiet and active sleep [159–161]. New work by van Swinderen and their team suggests that, in insects just like in mammals, active sleep serves a function of calibrating the brain as an optimal ‘prediction machine’ [71,162]. Local field potential (LFP) recordings during sleep show that periods of oscillatory activity during active sleep alternate with periods of decreased overall activity during quiet sleep [71,163]. Calcium imaging has confirmed that during these periods, different groups of neurons were recruited [164]. These discoveries have contributed clues to the functional role of these sleep stages and why they might have evolved, as well as some of their similarities to mammalian REM and deep sleep. In *Drosophila melanogaster*, the latter has been associated with cellular waste clearance and repair [165,166]. These processes are also present during deep sleep in mammals and suggest a basic evolutionary function to deep sleep: cellular homeostasis [167,168].

What is the function of active sleep in insects? Of course, there is no direct access to the question of whether insects dream (no more or less than any other animals). Interestingly, however, distinct theta oscillations are found to emanate from the central complex of the fly brain—an area associated with visual attention and navigation [163,169–172]. Some evidence also suggests that prediction drives sleep need and that recognition of unexpected stimuli (likely to produce a prediction error) is attenuated during active sleep [135,173]. Further understanding of the functional link between active sleep and predictive processing might also help to tie sleep in with other cognitive functions associated with the emergence of consciousness. A bi-directional interaction between sleep and attention has been proposed and responses to salient information have been explored in sleeping flies, where a circuit that modulates sensory perception has been found: fruit flies are more likely to wake when exposed to salient olfactory stimuli, a behavioural finding mediated by a connection between peripheral olfactory inputs and sleep regulatory neurons [140,174,175]. Closed-loop simulations of the kind required to optimize predictive models during sleep might also require whole-brain activations; recent work suggests that a similar process occurs during imagination, when signal strength in whole perceptual hierarchies distinguishes real and imagined stimuli [176].

Predictive processing has been the dominant mechanistic framework through which possible conscious phenomena have been explored in insects, even though it is not universally accepted that it is the most powerful framework for the exploration of conscious awareness [16,177]. Future work might seek to explore the plausibility of insect consciousness under different theories, such as the global workspace theory or integrated information theory [178,179].

## 6. Conclusion

We have focused here on an idiosyncratic set of topics in the broader field of the exploration of insect consciousness. We have not covered many other relevant cognitive lines of investigation in this context, because these have been covered for insects in detail elsewhere [180]. These include studies on intentionality [181], perceptual rivalry [182], foresight [183], flexible combination of past memories into concepts [184], cross-modal object recognition [185], interval timing and trace conditioning [186,187] and episodic-like memories [188,189], as well as perceptual binding [190]. None of these are, individually, formal demonstrations of consciousness, but further positive evidence from all these lines of investigation (ideally all in the same species) would add to the picture that we have assembled here on attention and prediction, its possible interaction with sleep and the question of emotions, as well as the distinction of self and other: they make the existence of conscious experience in insects at least plausible. Such cognitive processes are probably adaptive: for example, an awareness of one’s own body, the ability to trade off noxious stimuli with rewards or to efficiently react to uncertainty in the environment seem to proffer important fitness benefits.

A critical reader might observe that each of the behaviours and each of the neural processes described above could also be programmed into a non-conscious robot. An example is a review on animal pain by Sneddon *et al.* [191]. The authors hold that the same overtly observable phenomena that, in vertebrates, are typically taken as indicators of pain experiences, cannot be used as equally valid indicators in invertebrates—because, so the authors argue, robots can be manufactured to display similar phenomena. It is unclear why this argument should apply disproportionately more to one taxon than another. More broadly, we counter that nature has no room for such profligacy in generating beings that just pretend to ‘tick boxes’ in various paradigms designed to explore consciousness across animals. The robot analogy also overlooks that engineering products and evolutionarily acquired functions come about by entirely different pathways. They are subject to different developmental constraints, using entirely different building blocks and computations, and, in the engineering case, there is full knowledge of the functionality of the desired end-product. For example, the fact that it is technically possible to construct algorithms and machinery to recognize human faces tells us nothing about the neural mechanisms or evolutionary pathways underpinning human face recognition. Much less does the existence of such engineering products prove that biological face recognition works without conscious experience of seeing faces.

Importantly, if indeed we were to propose that insects achieve all of the phenomena described here by non-conscious processes, this would imply the following challenges. We would have to explain why the commonly cited adaptive benefits of consciousness in humans [192] can be mediated by fully automated mechanisms in non-human animals (and conversely, why they could not have been automated in humans [76]). Many of the common adaptive arguments regarding the purpose of humans consciousness would collapse: it is often argued that there is an essential role of emotions in learning, as well as in seeking rewarding conditions and avoiding danger, and also that attention is survival-related, that mental exploration is crucial in flexible problem-solving, etc. [193–195]. If insects exhibit all these phenomena, overtly displaying common *symptoms* of attention, emotion, flexible cognitive problem-solving without trial-and-error, etc., while at the same time lacking consciousness, then the adaptive need for consciousness for these phenomena in humans would be questionable as well.

The study of consciousness in insects, then, suggests functional stepping stones to the development of subjective experience, related cognitive functions and their implementation in the brain. The variety of selection pressures and lifestyles that the insect world offers is a perfect natural laboratory to further explore under which conditions consciousness might evolve. The relative compactness of the insect nervous system and existence of connectomes and brain-wide recording methods makes insects useful models of the circuitry-level investigation of conscious experience as well, in addition to the goal of identifying relevant selection pressures and plausible evolutionary scenarios for its implementation. As we grow to understand the specific binding of cognitive function and brain connectivity that produces subjective experience in humans, we will also get closer to drawing the line of where consciousness first arises.

## Data Availability

This article has no additional data.
